# First Indian report on genome-wide comparison of multidrug-resistant *Escherichia coli* from blood stream infections

**DOI:** 10.1371/journal.pone.0220428

**Published:** 2020-02-26

**Authors:** Naveen Kumar Devanga Ragupathi, Balaji Veeraraghavan, Dhiviya Prabaa Muthuirulandi Sethuvel, Shalini Anandan, Karthick Vasudevan, Ayyan Raj Neeravi, Jones Lionel Kumar Daniel, Sowmya Sathyendra, Ramya Iyadurai, Ankur Mutreja

**Affiliations:** 1 Department of Clinical Microbiology, Christian Medical College, Vellore, India; 2 Department of Chemical and Biological Engineering, The University of Sheffield, Sheffield, United Kingdom; 3 Department of Medicine, Christian Medical College, Vellore, India; 4 Department of Medicine, Addenbrookes Hospital, University of Cambridge, Cambridge, United Kingdom; 5 Translational Health Science and Technology Institute (THSTI), Delhi-NCR, India; Panstwowy Instytut Weterynaryjny - Panstwowy Instytut Badawczy w Pulawach, POLAND

## Abstract

**Background:**

Multidrug-resistant (MDR) *E*. *coli* with extended-spectrum β-lactamases (ESBLs) is becoming endemic in health care settings around the world. Baseline data on virulence and antimicrobial resistance (AMR) of specific lineages of *E*. *coli* circulating in developing countries like India is currently lacking.

**Methods:**

Whole-genome sequencing was performed for 60 MDR *E*. *coli* isolates. The analysis was performed at single nucleotide polymorphism (SNP) level resolution to identify the presence of their virulence and AMR genes.

**Results:**

Genome comparison revealed the presence of ST-131 global MDR and ST410 as emerging-MDR clades of *E*. *coli* in India. AMR gene profile for cephalosporin and carbapenem resistance differed between the clades. Genotypes *bla*_CTX-M-15_ and *bla*_NDM-5_ were common among cephalosporinases and carbapenemases, respectively. For aminoglycoside resistance, *rmtB* was positive for 31.7% of the isolates, of which 95% were co-harboring carbapenemases. In addition, the FimH types and virulence gene profile positively correlated with the SNP based phylogeny, and also revealed the evolution of MDR clones among the study population with temporal accumulation of SNPs. The predominant clone was ST167 (*bla*_NDM_ lineage) followed by ST405 (global clone ST131 equivalent) and ST410 (fast spreading high risk clone).

**Conclusions:**

This is the first report on the whole genome analysis of MDR *E*. *coli* lineages circulating in India. Data from this study will provide public health agencies with baseline information on AMR and virulent genes in pathogenic *E*. *coli* in the region.

## Introduction

*Escherichia coli* is the leading cause of bloodstream infections (BSIs) [1] and other common infections including urinary tract infections (UTIs). As an important commensal component of the biosphere, *E*. *coli* colonizes the lower gut of animals and humans and gets released in the environment.

Virulence of *E*. *coli* is driven by multiple factors including adhesins, toxins, siderophores, lipopolysaccharide (LPS), capsule, and invasins [2]. It has recently been reported that a large proportion of multi-drug resistant (MDR) *E*. *coli* carried by people is food acquired, especially from farm animals [3]. Although most of the MDR *E*. *coli* are reported to be community acquired, recently MDR *E*. *coli*, which produce extended-spectrum β-lactamases (ESBLs) have been found to be endemic in health care settings [4,5].

Among MDR *E*. *coli*, AMR caused by ESBL is mainly due to the *bla*_CTX-M_ family, particularly *bla*_CTX-M-15_ and _-14_, compared to the less frequently observed *bla*_SHV_ and *bla*_OXA_ families [6–8]. As per the literature, carbapenem resistance in *E*. *coli* is mostly mediated by *bla*_OXA-48_ [9], *bla*_NDM_ and *bla*_VIM_ genes [10]. Also, increasingly, resistance is being reported for fluoroquinolones and third- and fourth-generation cephalosporins and ST-131 predominates globally among such MDR *E*. *coli* strains [11].

This current study was aimed at identifying the predominant virulent and AMR genes in MDR *E*. *coli* circulating in India. Core genome phylogeny was constructed using high quality SNP profiles to analyse the genome wide factors associated with these genes in *E*. *coli* isolates analyzed or sequenced.

## Materials and methods

### Isolates and identification

A total of 99257 specimens were received at the Department of Clinical Microbiology, Christian Medical College, Vellore, India for routine screening from BSI during the year 2006 to 2016. Isolation and identification of the organism were carried out using a standard protocol as reported earlier [12]. Of the 1100 samples found culture positive for *E*. *coli*, 10% were resistant to carbapenems, of which 60 MDR isolates were selected for further characterization.

### Antimicrobial susceptibility testing (AST)

#### Disc diffusion

AST testing was carried out using the Kirby-Bauer disk diffusion method. The antimicrobial agents tested were Amikacin (30 μg), netilmicin (30 μg), gentamycin (10 μg), chloramphenicol (30 μg), ciprofloxacin (5 μg), cefotaxime (30 μg), cefoxitin (30 μg), ceftazidime (30 μg), cefpodoxime (10 μg), piperacilllin-tazobactam (100/10 μg), cefoperazone-sulbactam (75/30), imipenem (10 μg) and meropenem (10 μg), tigecycline (15 μg) and tetracycline (30 μg) according to guidelines suggested by Clinical and Laboratory Standards Institute (CLSI) M100-S27, 2017. Quality control strains (*K*. *pneumoniae* ATCC 700603, *P*. *aeruginosa* ATCC 27853 and *E*. *coli* ATCC 25922) were used in all batches, as per the CLSI recommendation.

#### Minimum Inhibitory Concentration (MIC) for colistin

Colistin MICs for the studied isolates were determined by broth microdilution and interpreted using CLSI 2017 breakpoint recommendations. *mcr-1* positive *E*. *coli* with the expected range 4–8 μg/ml, *E*. *coli* ATCC 25922 (0.25–2 μg/ml) and *P*. *aeruginosa* ATCC 27853 (0.5–4 μg/ml) were used as quality and technical control (QC and TC) strains for colistin MIC determination.

### Next generation sequencing and genome assembly

Genomic DNA was extracted using a QIAamp DNA Mini Kit (QIAGEN, Hilden, Germany). Whole genome sequencing (WGS) was performed using an Ion Torrent^™^ Personal Genome Machine^™^ (PGM) sequencer (Life Technologies, Carlsbad, CA) with 400-bp read chemistry according to the manufacturer’s instructions. Data were assembled with reference *E*. *coli* strain (NC000913) using Assembler SPAdes v.5.0.0.0 embedded in Torrent Suite Server v.5.0.3.

### Genome annotation

The assembled sequence was annotated using PATRIC, the bacterial bioinformatics database and analysis resource (http://www.patricbrc.org), and NCBI Prokaryotic Genomes Automatic Annotation Pipeline (PGAAP, http://www.ncbi.nlm.nih.gov/genomes/static/Pipeline.html). Downstream analysis was performed using the CGE server (http://www.cbs.dtu.dk/services) and PATRIC. The resistance gene profile was analysed using ResFinder 2.1 from the CGE server (https://cge.cbs.dtu.dk//services/ResFinder/). The sequences were also screened for antimicrobial resistance genes in the Antibiotic Resistance Genes Database (ARDB) and Comprehensive Antibiotic Resistance Database (CARD) through PATRIC. Virulence genes from the genomes were identified using VirulenceFinder 2.0 (https://cge.cbs.dtu.dk/services/VirulenceFinder/). Serotype of the isolates were identified using SerotypeFinder 1.1 (https://cge.cbs.dtu.dk/services/SerotypeFinder/).

### Genome based MLST analysis

Sequence types (STs) were analysed using multi-locus sequence typing (MLST) 1.8 tool (https://cge.cbs.dtu.dk//services/MLST/). To visualize the possible evolutionary relationships between isolates, STs of the study isolates and the globally reported strains were computed using PHYLOViZ software v2.0 based on goeBURST algorithm. The study used Warwick database for all sequence based MLST analysis of *E*. *coli*.

### Genome comparison analyses

Gview, interactive genome viewer was used to compare the annotated *E*. *coli* genome arrangements with the reference *E*. *coli* K12 genome (NC_000913) [13]. Core genome analysis was performed using Roary: the Pan Genome Pipeline v3.11.2 from Sanger Institute [14]. The phylogenetic tree was constructed using the core SNPs using FastTree v2.1.10. To evaluate the effect of recombination regions on the *E*. *coli* genomes, SNIPPY was performed to retrieve core SNPs, that was followed by Genealogies Unbiased By recomBinations In Nucleotide Sequences (Gubbins) algorithm [15]. The tree was constructed with midpoint rooting. Further, the tree file was visualised and analysed in iTOL v4 (https://itol.embl.de/). A dendrogram representing core vs pan genes was constructed using hierarchical cluster analysis with hclust method in R.

This Whole Genome Shotgun project has been deposited at GenBank under the accession numbers as mentioned in S1 Table. The version described in this manuscript is version 1.

### Ethical clearance

The study was approved by the Institutional Review Board and Ethical committee, Christian Medical College, Vellore, India (IRB No.: 9540 dt 22-07-2015). All the samples were fully anonymized before processing and since our study only utilised isolates received from routine blood cultures, we did not require informed written consent from the patients.

## Results

### Antimicrobial susceptibility

All 60 *E*. *coli* isolates were resistant to carbapenems, quinolones, cephalosporins and beta-lactamase inhibitors (S1 Table). Whereas all the isolates were susceptible to colistin except B7532 and B9021, which exhibited an MIC of 32 μg/ml.

### Whole genome sequence analysis

#### Phylogeny of MDR *E*. *coli*

MLSTFinder revealed the different sequence types of the isolates. The study isolates belonged to 6 clonal complexes with 14 different sequence types. Few of the sequence types were observed to share same founder types revealing the evolution of these strains. CC10 and CC 405 were the two major CCs observed with ST-167, ST-410 and ST-405 as the common STs. Interestingly, nine isolates belonging to CC/ST-131 were identified, of which, all were of H-30 clade, except the isolate BA9313 (H-24).

#### *E*. *coli* genome comparison

Whole genome composition of 60 MDR *E*. *coli* was compared with the *E*. *coli* K-12 reference genome which shows the region of differences between these genomes (S1 Fig). A total of 2,518,792 SNPs were identified in all the analyzed genomes. On minimum, 5957 and maximum, 74713 SNPs were identified in the study MDR *E*. *coli* genomes when compared to the reference genome.

### Core vs pan genome

**Fig 1:**
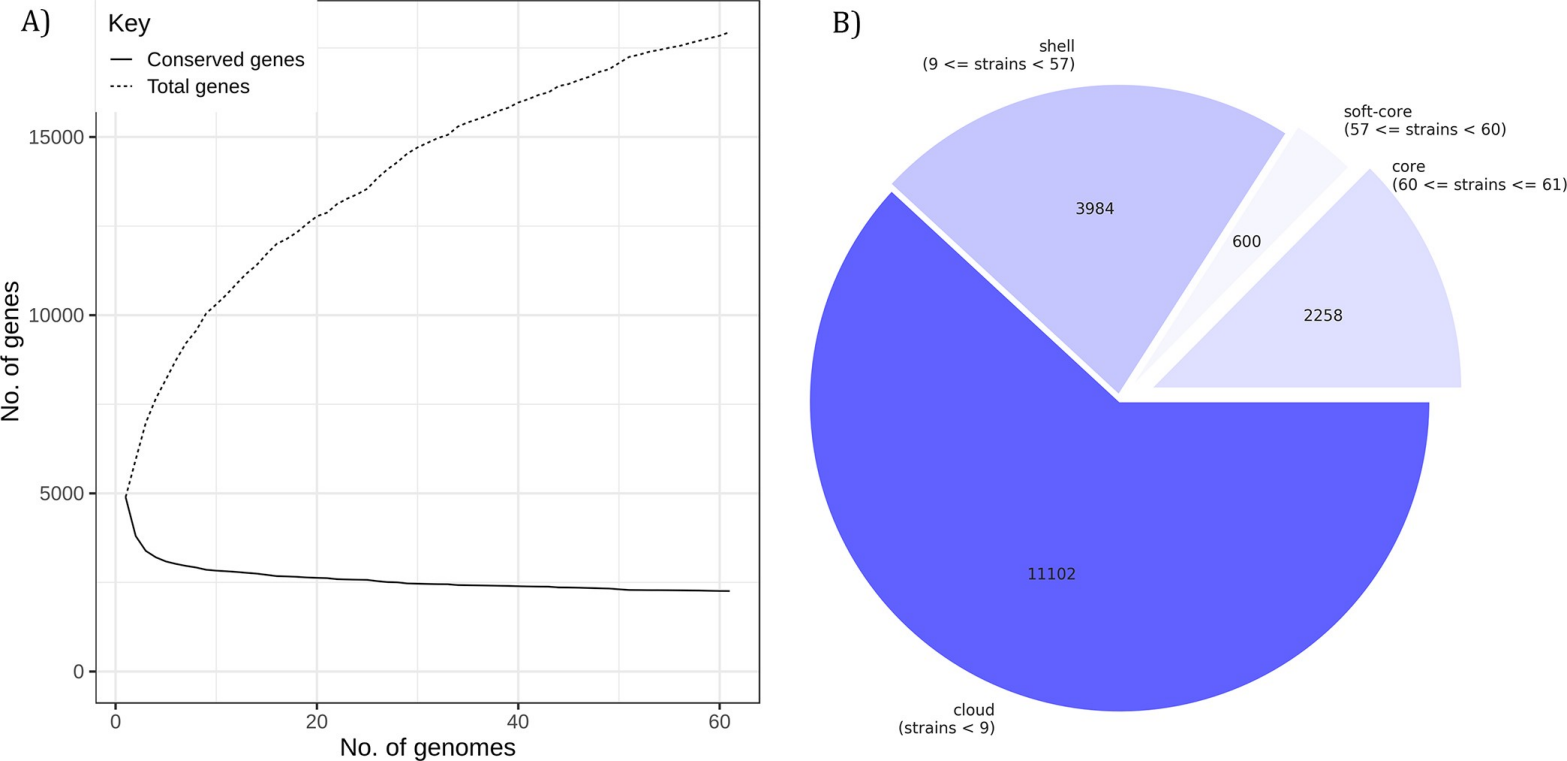
Pan genome vs core genome comparison depicting number of pan genes and conserved genes (A), Distribution of core genes, soft core genes and pan genes among the 60 MDR *E*. *coli* isolates from BSI (B).

Comparison between the core and pan genomes of 60 MDR *E*. *coli* isolates revealed 2258 core genes across all 60 isolates among the 17944 total gene clusters. This includes 600 soft core genes in 57 to 59 isolates, 3984 shell genes in 9 to 57 isolates and 11102 genes in less than 9 isolates (Fig 1).

#### Serotype prediction

Serotypes were established from the whole genome data and O102:H6 was the most common serotype (18.75%), followed by O89:H9 (15.6%), O8:H9 (12.5%), O89:H5 (9.4%) and other serotypes (S1 Table).

#### Genetic virulence factors of MDR *E*. *coli*

Three common virulence gene profiles were observed among the isolates as follows, i) *iss*, *capU*, *gad*, ii) *ipfA*, and iii) *eilA*, *gad*, *air*. The FimH virulence typing revealed the types 5, 24, 27, 28, 30, 35, 54, 191 and 54-like in comparison to the fimH database.

#### Comparison of virulence and clonal traits

**Fig 2:**
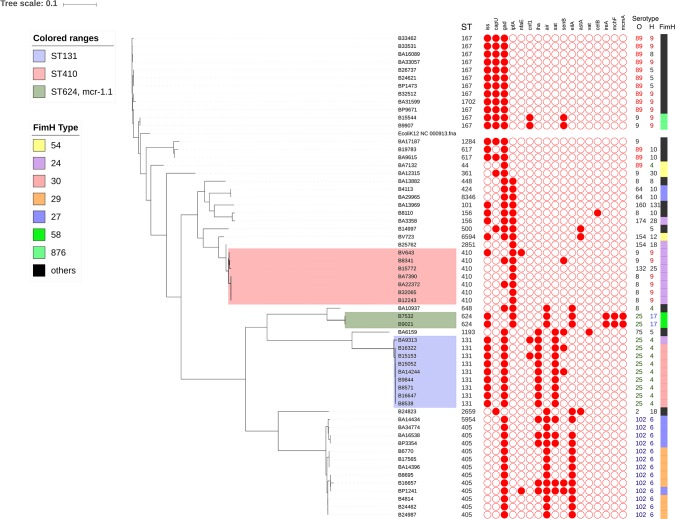
SNP phylogeny based comparison of genetic virulence traits observed in MDR *E*. *coli* strains. Sequence types, virulence gene profile, O and H antigens and Fim-H types are shown next to the tree.

The virulence gene profiles of the 60 isolates were compared to the FimH virulence types, MLST sequence type and SNP phylogeny. The isolates clustered in two distinct groups including ST-131(H-30 clade), based on the virulence genes identified (Fig 2). The sequence types were found to be tightly linked to the groups of virulence gene profile and FimH types.

#### Antimicrobial resistance genetic determinants

**Fig 3:**
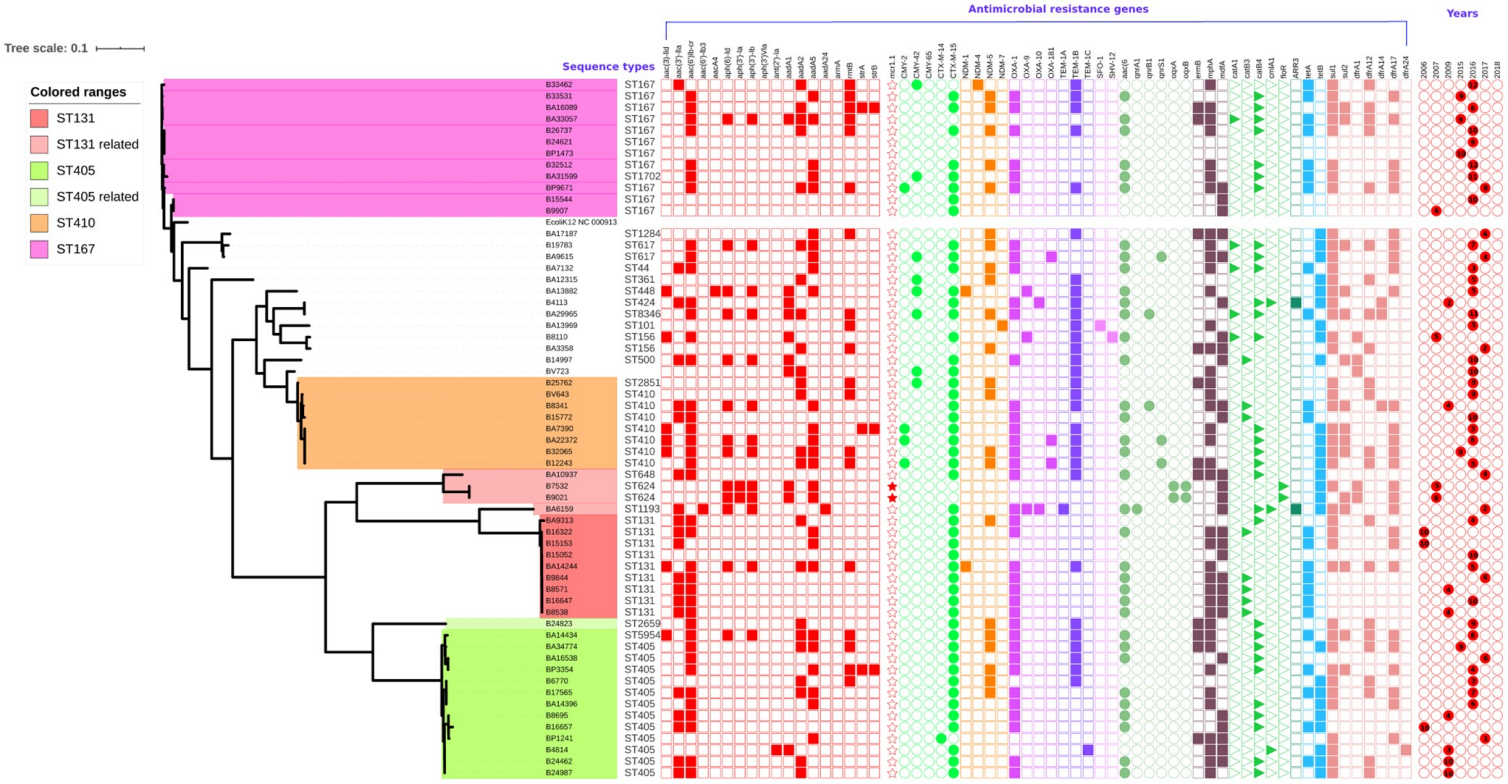
Antimicrobial resistance genes observed in MDR *E*. *coli* compared to the SNP based phylogeny. Depicting prevalence of *bla*_NDM-5_ among carbapenemases and *bla*_CTX-M-15_ among cephalosporinases.

ResFinder revealed the presence of multiple AMR genes in each of the MDR *E*. *coli* (Fig 3). Aminoglycoside and beta lactam resistance genes were the most dominant. The most common aminoglycoside resistance genes were *aadA5* and *aac(6’)lb-cr*, followed by *aadA2* and *rmtB*, while *bla*_CTX-M-15_ followed by *bla*_NDM-5_, *bla*_OXA-1_ and *bla*_TEM-1B_ were most prevalent among beta lactamases. Most of the isolates also harboured *mphA*, *catB4*, *sul1*, *tetB*, *dfrA17* and *dfrA12*. Interestingly, two isolates, B7532 and B9021 carried *mcr-1*.*1*, which is responsible for plasmid-mediated colistin resistance. The two isolates also showed phenotypic resistance to colistin with high MIC (>32 μg/ml). In addition, the phenotypic resistance for other antimicrobials exhibited significant correlation (>80%) with the presence of respective AMR genes.

## Discussion

The increasing use of third-generation β-lactams and β-lactam inhibitors was accompanied with increases in prevalence of the MDR phenotype among *E*. *coli*. The susceptibility profiles noted in invasive *E*. *coli* isolates of our study were similar to the previously (2014–2016) reported susceptibility to cefoxitin 53%, ceftazidime 33%, cefotaxime 26%, ceftriaxone 25%, cefepime 29%, piperacillin tazobactam 66%, imipenem and meropenem 89%, aztreonam 36%, ciprofloxacin 19%, levofloxavin 23%, and amikacin 91% [16]. Among these, about 64% of *E*. *coli* were found to be ESBL producers.

On genotypic characterization of MDR *E*. *coli* isolates, the increasing frequency of antimicrobial resistance in clinical *E*. *coli* isolates was found to be associated with *bla*_CTX-M_, *bla*_NDM_, and *mcr* genes. In our study, multiple AMR genes for beta lactams, carbapenems, fluoroquinolones, tetracycline, aminoglycosides and colistin were identified. The presence of genotypic AMR genes correlated well with phenotypic expression for beta lactams, carbapenems, fluoroquinolones and tetracycline. Plasmids IncFII majorly carried AMR genes *bla*_CTX-M-15_, *bla*_NDM-5_, *aadA2*, *rmtB*, *sul1*, *drfA12*, *erm*(B) and *tetA*, while IncFI plasmids carried mostly *aadA5*, *sul2*, *dfrA17*, *mph*(A) and *tetB* genes. Results from plasmid analysis of the study isolates were previously published elsewhere [17].

The MDR *E*. *coli* isolates phylogenetically grouped into four major clades: ST167, ST410, ST405 and ST131. Variant *bla*_NDM-5_, responsible for carbapenem resistance was common in comparison to other *bla*_NDM_ variants. The *bla*_NDM_ positive isolates belonging to ST410 and ST405 harboured only the *bla*_NDM-5_ variant, whereas ST167 and ST131 isolates had *bla*_NDM-4_ and *bla*_NDM-1_ respectively, in addition to *bla*_NDM-5_. Interestingly only two isolates out of 60 MDR *E*. *coli* had *bla*_NDM-1_, while it is still common among other species of MDR clinical pathogens in India [18]. From Hong Kong, a previous report identified *bla*_NDM-1_ as common among *E*. *coli* though the sample size was lesser [19], whereas in central China, *bla*_NDM-1_ and *bla*_NDM-5_ occurrence in *E*. *coli* has been reported in equal numbers [20].

Globally, *bla*_OXA-48_ type were the most commonly reported carbapenemases among *E*. *coli* [21], followed by *bla*_NDM_ [22], *bla*_IMP_ [23] and *bla*_KPC_ [24]. Studies have reported occurrence of *bla*_OXA-48_ from as low as 3% to 22% [25, 9]. In contrast, a previous report from India on carbapenem-resistant clinical *E*. *coli* isolates from 2013 and 2015 has shown that *bla*_NDM_ was common among carbapenemases in *E*. *coli* (70%), followed by *bla*_OXA-48_ (24%) and *bla*_VIM_ (17%). Co-occurences of *bla*_NDM_ along with *bla*_OXA_ (5%) and *bla*_VIM_ (17%) have also been reported [26]. Similar results were seen in our study and the combinations observed were only *bla*_NDM_+*bla*_OXA-1_. Though, *bla*_OXA-181_ was rare in combination with *bla*_NDM_ (*n* = 1). These observations confirm that *bla*_NDM_ is prevalent among *E*. *coli* followed by *bla*_OXA_ in India, which is otherwise the most prevalent elsewhere.

There has been a global concern on aminoglycoside resistance in Gram-negatives. Acquired 16S-RMTases are known to confer extremely high level of aminoglycoside resistance, due to which key aminoglycosides including gentamicin, tobramycin, and amikacin are ineffective against carbapenem resistant strains [27]. Accordingly, plazomicin, a new aminoglycoside agent identified to combat against carbapenem-resistant Enterobacteriaceae, was found inactive if the isolates co-produced 16S-RMTases [28]. In this study, ~ 95% of the RMTase positive *E*. *coli* co-harboured carbapenemases, which worryingly contributes to the already high burden of carbapenem resistance. Similar to our study, Taylor et al. [29] and Poirel et al. [30] have reported 83.1% and 45.4% co-occurrence of carbapenemases in 16S RMTase producing Enterobacteriaceae, respectively.

Our study shows that, for cephalosporin resistance, the isolates from ST131 and ST405 clades carried *bla*_CTX-M-15_ in 100% and 92% of their respective clades, whereas ST167 and ST410 isolates carried 18% and 43% *bla*_CMY_ genes in addition to 63% and 100% *bla*_CTX-M-15_. However, 54.34% *bla*_CTX-M_ was reported previously in ESBL positive isolates from India [31]. Among the study isolates, ST167 carried significantly (P<0.05) lesser *bla*_CTX-M-15_ in comparison to other clades. Similarly, ST167 and ST410 carried *bla*_CMY_ in addition for cephalosporin resistance which was not seen in ST131 and ST405. Recently, plasmid-mediated colistin resistance is being increasingly reported in *E*. *coli* [32–34]. This study also observed two isolates (B7532, B9021) with *mcr-1*.*1* expressing high MIC of >32 μg/ml to colistin and both the isolates, from the same time period and ward, were closely related with same sequence type (ST624). After this observation made in 2007 strains, there have been no reports of *mcr*.

The antimicrobial susceptibility of *E*. *coli* has been shown to vary geographically [35]. Among the different clonal groups observed elsewhere, *E*. *coli* ST131 was previously reported to be most commonly associated with community acquired infection [36–37], which recently were highly associated with healthcare settings. Also, ST131 was reported earlier as the predominant lineage carrying *bla*_CTX-M-15_ and other ESBLs. Most of the MDR *E*. *coli* carrying *bla*_CTX-M-15_ from different countries in Europe and North America were homogenously grouped into the *E*. *coli* O25:H4-ST131 [6,36–37]. In our study, 87% of the isolates carried *bla*_CTX-M-15_, among various STs, with only nine isolates of ST131. Among the observed STs in this study, *bla*_CTX-M-15_ was previously reported for its association with ST617, ST405 and ST131 [37].

Though ST131 clones were predominantly reported worldwide, the STs observed in our study were striking for clustering in distinct phylogenetic lineages. ST167 was previously reported for its ability to carry *bla*_NDM_ genes in China [38–40]. ST405 has been known as another global clonal group similar to ST131 [41] and has been reported to carry *bla*_NDM_ genes in hospital settings [42], in addition to *bla*KPC-2 [43]. ST405 was reported as a lineage, carrying fluoroquinolone resistance in Japan [41]. Recently, ST410 was reported as a possible international high risk clone with B2/H24R, B3/H24Rx, and B4/H24RxC AMR clades. B3/H24Rx was reported to be evolved by acquisition of the *bla*_CTX-M-15_ and an IncFII plasmid. B4/H24RxC emerged by acquiring IncX3 plasmid with *bla*_OXA-181_ known for carbapenem resistance, which further acquired *bla*_NDM-5_, on a conserved IncFII plasmid [44]. In this study, all ST410 isolates (*n* = 7) harboured *bla*_CTX-M-15_ gene, while only B25762, BV643, B12243 and B32605 had IncFII plasmids and *bla*_NDM-5_ (B3/H24RxC). B12243, in addition harboured IncX3 with *bla*_OXA-181_ (B4/H24RxC), while BA22372 and BA9615 had only *bla*_OXA-181_ in IncX3 plasmid (B4/H24RxC).

Virulence genes observed among the *E*. *coli* isolates varied according to the different clades observed. The comparison of the virulence gene type with SNP based phylogeny revealed the acquisition and deletion of virulence genes. Genes *iss*, *capU* and *gad* were observed in ST167 clade. ST131 possessed *iha*, *sat*, *cnfl* and *senB*, in addition to *iss* and *gad*. ST131 strains in our study have lost the *capU* genes. Further, ST405 clade also lost *iss* and gained *eilA* and *air* genes with FimH type 29. Few isolates of ST405 retained *iha* and *sat* genes belonging to FimH 27 type within ST405. ST410 (FimH 24) that predominantly had *ipfA* gene, on the contrary, lost all other genes, except *gad* gene in two isolates. Overall, *gad* gene served as backbone for ST167, ST131 and ST405 clades, while *ipfA* was consistent in ST410. Ours is the first study that compares the evolution of virulence pattern with phylogeny, which explains the emergence of a stable clinical virulent phenotype.

FimH, that had been reported as a major candidate for the development of a vaccine against pathogenic *E*. *coli* [45] is responsible for producing mannose-sensitive bacterial adhesion [45]. Though high nucleotide conservation of >98% was observed in *fimH* alleles, minor sequence differences have been reported to correlate with differential binding and adhesion phenotypes [46]. Fim-H types in our study correlated well with the STs.

Our study shows that with a SNPs based phylogeny, higher discrimination between the clinical MDR *E*. *coli* isolates is apparent. Therefore, more such studies with integrated approach to analysing pathogenic *E*. *coli* in India are required to fully understand and follow the dynamic virulence and AMR landscape of this rapidly evolving group of pathogens.

## Conclusions

To the best of our knowledge, this is the first report on SNP phylogeny in comparison with AMR and virulence traits in *E*. *coli* in India. The study revealed the prevalence of *bla*_NDM-5_ among the clades ST131, ST405 and ST410 clades. *bla*_CTX-M-15_ was responsible for cephalosporin resistance in ST131 and ST405 clades whereas, ST167 and ST410 carried both *bla*_CTX-M-15_ and *bla*_CMY_ genes. For aminoglycoside resistance, *rmtB* was positive for 31.7% of the isolates, of which 30% were co-harbouring carbapenemases. The FimH types and virulence gene profile positively correlated with the SNP based phylogeny. However the predominant ST131 epidemic clone was smaller in our study population while ST167 and ST405 clones with multiple AMR genes were predominant. Further larger studies are needed to rule out any possible bias. Isolates with *iss*, *capU* and *gad* virulence genes were the major type. Moreover, SNP based phylogeny revealed evolution of the MDR clones among the study population, which suggests that continuous WGS level molecular surveillance would be necessary to keep track of the spread of MDR clones in India.

## Supporting information

S1 Table Serotype and antimicrobial resistance profiles of MDR *E*. *coli* from blood stream infections (*n* = 60). (DOCX)

S1 Fig Circular genome plot comparing 60 MDR *E*. *coli* thereby showing differences in genome composition in comparison to the reference genome NC000913 *E*. *coli*. (TIF)
