# Supplementary material for: First Indian report on genome-wide comparison of multidrug-resistant Escherichia coli from blood stream infections
Source: PLoS One. 2020 Feb 26;15(2):e0220428. doi: 10.1371/journal.pone.0220428 (PMC7043739; doi:10.1371/journal.pone.0220428)
Supplement: S1 Table — (DOCX) [file pone.0220428.s001.docx]

**Table S1:** Serotype and antimicrobial resistant profiles of MDR *E. coli* from blood stream infections. CTX-cefotaxime; CZD-ceftazidime; CPD-cefpodoxime; G-gentamicin; AK-amikacin; NET-netilmicin; CIP-ciprofloxacin; P/T-piperacillin-tazobactam; MEG-cefoperazone-sulbactam; IMI-imipenem; MEM-meropenem; CEF-cefoxitin; CPI-cefepime; MINO-minocycline; TET-tetracycline; CHL-chloramphenicol

| **S. no** | **Isolate** | **Serotype** | **Resistant pattern** | **Accession numbers** |
| --- | --- | --- | --- | --- |
| 1 | BA16089 | O89:H8 | CTX-CZD-CPD-G-AK-NET-CIP-P/T-MEG-IMI-MERO-CEF-CPI | PVPX00000000 |
| 2 | B26737 | O89:H5 | CTX-CZD-CPD-G-AK-NET-CIP-P/T-MEG-IMI-MERO-CEF-CPI-TET | PVPW00000000 |
| 3 | BA33057 | O89:H9 | CTX-CZD-CPD-G-AK-NET-CIP-P/T-MEG-IMI-MERO-CEF-CPI-TET | PVPV00000000 |
| 4 | B24621 | O89:H5 | CTX-CZD-CPD-G-AK-NET-CIP-P/T-MEG-IMI-MERO-CEF-CPI | PVPU00000000 |
| 5 | BP1473 | O89:H5 | CTX-CZD-CPD-G-AK-NET-CIP-P/T-MEG-IMI-MERO-CEF-CPI-TET | PVPT00000000 |
| 6 | B33462 | O89:H9 | CTX-CZD-CPD-G-AK-NET-CIP-P/T-MEG-IMI-MERO-CEF-CPI-TET | PVPS00000000 |
| 7 | B33531 | O89:H9 | CTX-CZD-CPD-G-AK-NET-CIP-P/T-MEG-IMI-MERO-CEF-CPI-TET | PVPR00000000 |
| 8 | B32512 | O89:H9 | CTX-CZD-CPD-CIP-P/T-MEG-IMI-MERO-CEF-CPI-TET | PVPQ00000000 |
| 9 | BA7132 | O89:H4 | CTX-CZD-CPD-G-CIP-P/T-MEG-IMI-MERO-CEF-CPI-TET-CHL | PVPP00000000 |
| 10 | BA31599 | O89:H9 | CTX-CZD-CPD-CIP-P/T-MEG-IMI-MERO-CEF-CPI | PVPO00000000 |
| 11 | B19783 | O89:H10 | CTX-CZD-CPD-CIP-P/T-MEG-IMI-MERO-CEF-CPI-MINO-TET-CHL | PVPN00000000 |
| 12 | BA7390 | O8:H9 | CTX -CZD -CPD- G-CIP-P/T-MEG-IMI-MERO-CEF-CPI-MINO-TET-CHL | PVPM00000000 |
| 13 | B12243 | O8:H9 | CTX-CZD-CPD-G-AK-NET-CIP-P/T-MEG-IMI-MERO-CEF-CPI-MINO-TET | PVPL00000000 |
| 14 | BA22372 | O8:H9 | CTX-CZD-CPD-G-CIP-P/T-MEG-IMI-MERO-CEF-CPI-MINO-TET | PVPK00000000 |
| 15 | BV643 | H9 | CTX-CZD-CPD-G-AK-NET-CIP-P/T-MEG-IMI-MERO-TIGI-CEF-CPI | PVPJ00000000 |
| 16 | B32065 | O8:H9 | CTX-CZD-CPD-G-AK-NET-CIP-P/T-MEG-IMI-MERO-CEF-CPI-TET | PVPI00000000 |
| 17 | B25762 | O154:H18 | CTX-CZD-CPD-G-AK-NET-CIP-P/T-MEG-IMI-MERO-CEF-CPI-CHL | PVPH00000000 |
| 18 | BA13882 | *O8:H8 | CTX-CZD-CPD-G-AK-NET-CIP-P/T-MEG-IMI-MERO-CEF-CPI-MINO-TET | PVOS00000000 |
| 19 | BA9313 | O25:H4 | CTX-CZD-CPD-G-CIP-P/T-MEG-IMI-MERO-CEF-CPI | PVPG00000000 |
| 20 | BA14244 | O25:H4 | CTX-CZD-CPD-G-AK-NET-CIP-P/T-MEG-IMI-MERO-CEF-CPI-TET | PVPF00000000 |
| 21 | BA14396 | *O102:H6 | CTX-CZD-CPD-CIP-P/T-MEG-IMI-MERO-CEF-CPI-MINO-TET | PVPE00000000 |
| 22 | B17565 | *O102:H6 | CTX-CPD-CZD-G-AK-NET-CIP-P/T-MEG-IMI-MERO-CEF-CPI-MINO-TET-CHL | PVPD00000000 |
| 23 | B6770 | *O102:H6 | CTX-CZD-CPD-G-AK-NET-CIP-P/T-MEG-IMI-MERO-CEF-CPI-MINO-TET | PVPC00000000 |
| 24 | BP3354 | O102:H6 | CTX-CZD-CPD-G-AK-NET-CIP-P/T-MEG-IMI-MERO-CEF-CPI-TET | PVPB00000000 |
| 25 | BA34774 | O102:H6 | CTX-CZD-CPD-G-AK-NET-CIP-P/T-MEG-IMI-MERO-CEF-CPI | PVPA00000000 |
| 26 | BA14434 | *O102:H6 | CTX-CZD-CPD-G-AK-NET-CIP-P/T-MEG-IMI-MERO-CEF-CPI-MINO-TET-CHL | PVOZ00000000 |
| 27 | B24823 | *O2:H18 | CTX -CZD -CPD- G- AK -NET-CIP-P/T-MEG-IMI-MERO-CEF-CPI-MINO-TET | PVOY00000000 |
| 28 | BA13969 | O160:H131 | CTX-CZD-CPD-G-AK-NET-CIP-P/T-MEG-IMI-MERO-CEF-CPI-MINO-TET | PVOX00000000 |
| 29 | BA12315 | O9:H30 | CTX-CZD-CPD-CIP-P/T-MEG-IMI-MERO-CEF-CPI-TET | PVOW00000000 |
| 30 | BA29965 | O64:H10 | CTX-CZD-CPD-G-AK-NET-CIP-P/T-MEG-IMI-MERO-CEF-TET-CHL | PVOV00000000 |
| 31 | BV723 | *O154:H12 | CTX-CZD-CPD-CIP-P/T-MEG-IMI-MERO-CEF-CPI | PVOT00000000 |
| 32 | BP9671 | O89:H9 | CTX-CZD-CPD-GEN-AMK-NET-CIP-PTZ-IMI-MERO-FOX-CPI | RCAC00000000 |
| 33 | BA17187 | O9: | CTX-CZD-CPD-GEN-AMK-NET-CIP-PTZ-CFB/SUL-IMI-MERO-FOX-CPI-TET | RCAE00000000 |
| 34 | BA6159 | O75:H5 | CTX-CZD-CPD-GEN-AMK-NET-CIP-PTZ-CFB/SUL-IMI-MERO-FOX-CPI-MIN-TET | RCAI00000000 |
| 35 | BA9615 | O162, O89:H10 | CTX-CZD-CPD-CIP-PTZ-CFB/SUL-IMI-MERO-FOX-MIN-TET | RCAH00000000 |
| 36 | BA3358 | O174:H28 | CTX-CZD-CPD-GEN-AMK-NET-CIP-PTZ-CFB/SUL-IMI-MERO-FOX-CPI | RCAJ00000000 |
| 37 | BP1241 | O102:H6 | CTX-CZD-CPD-GEN-AMK-NET-CIP-PTZ-CFB/SUL-IMI-MERO-FOX-CPI-TET-CHL | RCAD00000000 |
| 38 | BA10937 | O8:H4 | CTX-CZD-CPD-GEN-CIP-PTZ-CFB/SUL-IMI-MERO-FOX-CPI-MIN-TET | RCAG00000000 |
| 39 | BA16538 | O102:H6 | CTX-CZD-CPD-CIP-PTZ-CFB/SUL-IMI-MERO-FOX-CPI-MIN-TET | RCAF00000000 |
| 40 | B8110 | O40, O8:H10 | CTX-CZD-CPD-GEN-CIP-FOX-CPI-MIN-TET-CHL | RCAL00000000 |
| 41 | B4113 | O64:H10 | CTX-CPD-CZD-GEN-CIP-PTZ-CPI-MIN-TET-CHL | RCAN00000000 |
| 42 | B4814 | O102:H6 | CTX-CZD-CPD-GEN-CIP-PTZ-FOX-CPI-MIN-TET-CHL | RCAM00000000 |
| 43 | B24462 | O102:H6 | CTX-CZD-CPD-CIP-PTZ-CFB/SUL-FOX-CPI-TET | RCAK00000000 |
| 44 | B8695 | O102:H6 | CTX-CZD-CPD-GEN-CIP-FOX-CPI-MIN-TET | SAZJ00000000 |
| 45 | B15153 | O25:H4 | CTX-CZD-CPD-GEN-CIP-CPI-TET | SAZP00000000 |
| 46 | B16657 | O102:H6 | CTX-CZD-CPD-GEN-CIP-FOX-CPI-MIN-TET | SAZU00000000 |
| 47 | B24987 | O102:H6 | CTX-CZD-CPD-CIP-CPI-TET | SAZV00000000 |
| 48 | B7532 | O25:H17 | CIP-CPI-TET-CHL | SAZF00000000 |
| 49 | B8341 | H9 | CTX-CZD-CPD-GEN-CIP-CEF-CPI-TET | SAZG00000000 |
| 50 | B8538 | O25:H4 | CTX-CZD-CPD-GEN-CIP-PTZ-CEF-CPI-TET-CHL | SAZH00000000 |
| 51 | B8571 | O25:H4 | CTX-CZD-CPD-GEN-NET-CEF-CPI-TET-CIP | SAZI00000000 |
| 52 | B9021 | O25:H17 | CIP-TIG-MIN-TET-CHL | SAZK00000000 |
| 53 | B9844 | O25:H4 | CTX-CZD-CPD-GEN-CIP-CEF-TET | SAZL00000000 |
| 54 | B9907 | O9:H9 | CTX-CZD-CPD-GEN-AMK-CIP-CEF-CPI | SAZM00000000 |
| 55 | B14997 | H5 | CTX-CZD-CPD-GEN-CIP-CEF-CPI-MIN-TET-CHL | SAZN00000000 |
| 56 | B15052 | O25:H4 | CTX-CZD-CPD-GEN-AMK-CIP-CEF-CPI-CHL | SAZO00000000 |
| 57 | B15544 | O9:H9 | CTX-CZD-CPD-CIP-CEF-CPI | SAZQ00000000 |
| 58 | B15772 | O132:H25 | CTX-CZD-CPD-GEN-CIP-TIG-CEF-CPI-MIN-TET-CHL | SAZR00000000 |
| 59 | B16322 | O25:H4 | CTX-CZD-CPD-GEN-CIP-CPI-TET | SAZS00000000 |
| 60 | B16647 | O25:H4 | CTX-CZD-CPD-GEN-AMK-NET-CIP-CEF-CPI-TET | SAZT00000000 |

* Either O or H antigen genes did not show 100% coverage, needs to be further confirmed
