# Supplementary figures and images for: First Indian report on genome-wide comparison of multidrug-resistant Escherichia coli from blood stream infections
Source: PLoS One. 2020 Feb 26;15(2):e0220428. doi: 10.1371/journal.pone.0220428 (PMC7043739; doi:10.1371/journal.pone.0220428)

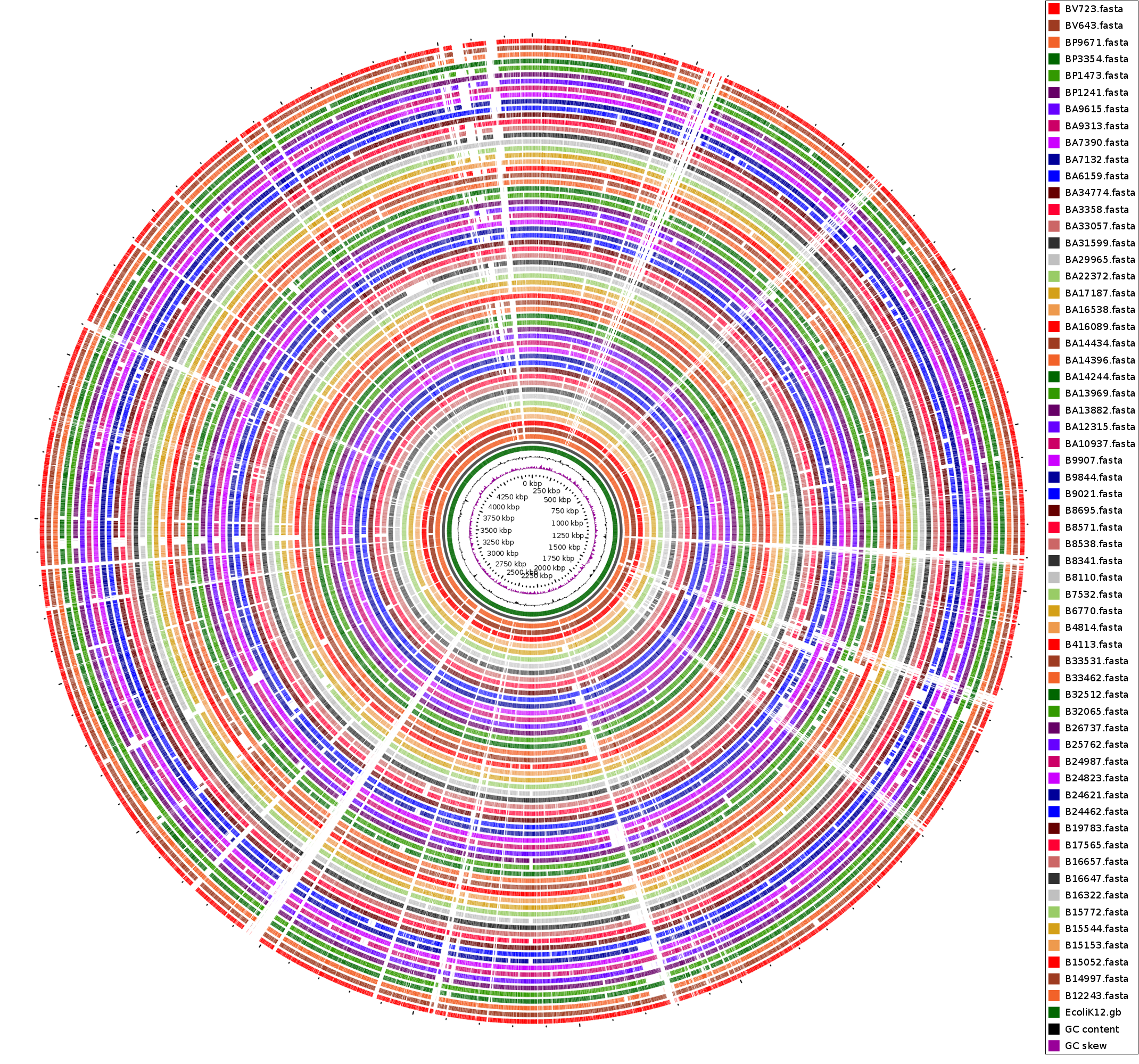

Supplement: S1 Fig — (TIF) [file pone.0220428.s002.tif]
